# Comparing Ventricular Synchrony in Left Bundle Branch and Left Ventricular Septal Pacing in Pacemaker Patients

**DOI:** 10.3390/jcm10040822

**Published:** 2021-02-17

**Authors:** Luuk I.B. Heckman, Justin G.L.M. Luermans, Karol Curila, Antonius M.W. Van Stipdonk, Sjoerd Westra, Radovan Smisek, Frits W. Prinzen, Kevin Vernooy

**Affiliations:** 1 Department of Physiology, Cardiovascular Research Institute Maastricht (CARIM), Maastricht University, 6200 MD Maastricht, The Netherlands; frits.prinzen@maastrichtuniversity.nl; 2 Department of Cardiology, Cardiovascular Research Institute Maastricht (CARIM), Maastricht University Medical Centre + (MUMC+), 6229 HX Maastricht, The Netherlands; justin.luermans@mumc.nl (J.G.L.M.L.); twan.van.stipdonk@mumc.nl (A.M.W.V.S.); kevin.vernooy@mumc.nl (K.V.); 3 Department of Cardiology, Radboud University Medical Centre (Radboudumc), 6526 GA Nijmegen, The Netherlands; Sjoerd.Westra@radboudumc.nl; 4 Department of Cardiology, Third Faculty of Medicine, Charles University and University Hospital Kralovske Vinohrady, 10034 Prague, Czech Republic; curilakarol@seznam.cz; 5 Institute of Scientific Instruments, the Czech Academy of Sciences, 61264 Brno, Czech Republic; rasmis@seznam.cz; 6 Department of Biomedical Engineering, Faculty of Electrical Engineering and Communication, Brno University of Technology, Technická 12, 61600 Brno, Czech Republic

**Keywords:** left bundle branch area pacing, bradycardia pacing, cardiac resynchronization therapy

## Abstract

Background: Left bundle branch area pacing (LBBAP) has recently been introduced as a novel physiological pacing strategy. Within LBBAP, distinction is made between left bundle branch pacing (LBBP) and left ventricular septal pacing (LVSP, no left bundle capture). Objective: To investigate acute electrophysiological effects of LBBP and LVSP as compared to intrinsic ventricular conduction. Methods: Fifty patients with normal cardiac function and pacemaker indication for bradycardia underwent LBBAP. Electrocardiography (ECG) characteristics were evaluated during pacing at various depths within the septum: starting at the right ventricular (RV) side of the septum: the last position with QS morphology, the first position with r’ morphology, LVSP and—in patients where left bundle branch (LBB) capture was achieved—LBBP. From the ECG’s QRS duration and QRS morphology in lead V1, the stimulus- left ventricular activation time left ventricular activation time (LVAT) interval were measured. After conversion of the ECG into vectorcardiogram (VCG) (Kors conversion matrix), QRS area and QRS vector in transverse plane (Azimuth) were determined. Results: QRS area significantly decreased from 82 ± 29 µVs during RV septal pacing (RVSP) to 46 ± 12 µVs during LVSP. In the subgroup where LBB capture was achieved (*n* = 31), QRS area significantly decreased from 46 ± 17 µVs during LVSP to 38 ± 15 µVs during LBBP, while LVAT was not significantly different between LVSP and LBBP. In patients with normal ventricular activation and narrow QRS, QRS area during LBBP was not significantly different from that during intrinsic activation (37 ± 16 vs. 35 ± 19 µVs, respectively). The Azimuth significantly changed from RVSP (−46 ± 33°) to LVSP (19 ± 16°) and LBBP (−22 ± 14°). The Azimuth during both LVSP and LBBP were not significantly different from normal ventricular activation. QRS area and LVAT correlated moderately (Spearman’s *R* = 0.58). Conclusions: ECG and VCG indices demonstrate that both LVSP and LBBP improve ventricular dyssynchrony considerably as compared to RVSP, to values close to normal ventricular activation. LBBP seems to result in a small, but significant, improvement in ventricular synchrony as compared to LVSP.

## 1. Introduction

When animal studies demonstrated that normal left ventricular (LV) function was preserved during pacing of the left side of the interventricular septum (IVS) [1], this so-called left ventricular septal pacing (LVSP) was applied for the first time in humans using the same transvenous approach through the IVS [2] Subsequently, Huang et al. demonstrated that, with a similar approach, it was feasible to capture the left conduction system by direct stimulation of the left bundle branch (LBB) resulting in a normal LV activation [3]. However, there seems to be a considerable overlap between LVSP and left bundle branch pacing (LBBP). Reported LBB capture success rates vary from 60% to 90% [4,5], and, consequently, up to one-third of patients are paced without LBB capture.

Despite the increasing number of publications on LBBP, many unknowns remain, such as the electrophysiological differences between LVSP and LBBP. A recently proposed measure for electrical dyssynchrony is the QRS area [6]. This three-dimensional QRS area expresses non-opposed electrical forces, and high values of this parameter indicate dyssynchronous electrical activation, even independent of the QRS morphology [7]. QRS area has also been shown to have a strong association with clinical and echocardiographic response to Cardiac Resynchronization Therapy (CRT) [8].

It was the aim of the present study to explore the electrophysiological changes in the course of the pacing lead penetrating the IVS. To this purpose, we evaluated QRS duration, QRS morphology, and QRS area in patients undergoing LBBP implantation during right ventricular (RV) pacing, deep LVSP, and LBBP.

## 2. Methods

### 2.1. Patient Selection

Patients suffering from symptomatic bradycardia without heart failure with an indication for pacemaker implantation underwent LBBP at the Maastricht University Medical Center (MUMC+) and at the Department of Cardiology of University Hospital Kralovske Vinohrady in Prague, Czech Republic, after informed written consent was obtained. The study was approved by the local ethics committee (Netherlands: METC 2019–1313, Czech Republic: EK-VP/06/0/2020).

### 2.2. Implantation Procedure

Pacemaker implantation with LBBP was performed as described previously [9]. In short, the right atrial (RA) lead was implanted according to routine clinical practice. Subsequently, the ventricular pacing lead (Medtronic 3830 lead) was inserted through the C315 His-sheath. An intracardiac electrogram was recorded from the lead tip using the electrophysiological recording system (Bard Electrophysiology Lab System, Marlborough, MA, USA). The His bundle electrogram was identified in the right anterior oblique (RAO) 20–25° position, and fluoroscopic image of the lead position was recorded as a reference. Subsequently, the sheath and the lead were advanced 1–2 cm toward the RV apex. In this region, unipolar pacing was performed, aiming for a paced QRS morphology with a notch in the nadir in lead V1. At this site, the lead was fixed in the RV septum with 1–2 rotations and then advanced to the left side of the IVS. In the process of advancing the pacing lead, fluoroscopic image and pacing parameters and morphologies were monitored to avoid displacement of the lead or perforation of IVS. 

**Figure 1:**
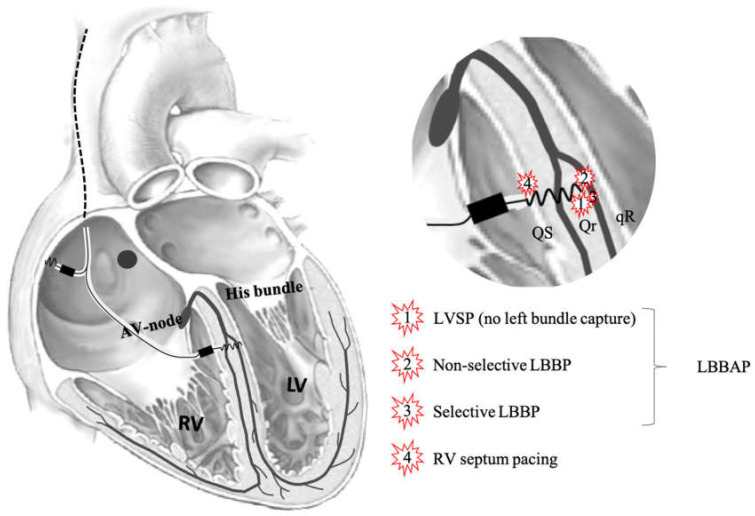
Schematic overview of the heart and the conduction system. Illustrated is where the lead penetrates the interventricular septum, pacing definitions are clarified, and shown is which QRS morphologies are typically seen. LVSP—left ventricular septum pacing. LBBP—left bundle branch pacing. LBBAP—left bundle branch area pacing. RV—right ventricle.

When advancing from right to left through the IVS, local electrogram from the lead tip, as well as paced 12-lead ECGs, were recorded after each rotation resulting in advancement of the lead. The number of attempts to implant the lead in the IVS, as well as the final position, were left to the implanting cardiologist. Capture of the left bundle was attempted in all patients. IVS pacing locations and definitions are depicted in Figure 1.

### 2.3. Pacing and Capture Definitions

RV septal pacing (RVSP) was defined as pacing with the lead tip at the RV septum before rotations were performed. Left bundle branch area pacing (LBBAP) was defined as the final position of the lead in all patients combined (no discrimination between LVSP and LBBP). 

LBB capture was defined as: (1) paced (pseudo) right bundle branch block (RBBB) QRS morphology with terminal r/R’ in lead V1, (2) recording of a LBB potential during intrinsic rhythm (only in patients with normal ventricular activation), (3) constant left ventricular activation time (LVAT) during high (8 V) and low (2 V) pacing output, and (4) the demonstration of transition from non-selective to selective LBBP (sLBBP) or non-selective LBBP (nsLBBP) to LV myocardial only capture during decreasing pacing output. sLBBP was defined as a change in QRS morphology without a change in S-LVAT during decreasing the pacing output from nsLBBP combined with an isoelectric interval between pacing spike and QRS complex (pacing spike distinct from ventricular electrocardiogram (EGM). nsLBBP was defined as a change in QRS morphology which occurred after increasing the pacing output from sLBBP or LVSP.

LVSP was defined as the last position of the lead before capture of the left conduction system (as defined previously) with r’ present in lead V1.

An R-wave with smaller amplitude compared to the preceded Q-wave is defined as r’ (either Qr morphology or Rsr’ morphology). R’ is defined as R-wave with larger amplitude compared to preceding Q-wave (either qR morphology or rsR’ morphology).

### 2.4. Electrical Measurements

Twelve-lead ECGs during pacing were recorded after each set of rotations resulting in advancement of the lead. After the procedure, these ECGs were assessed on QRS duration and morphology, especially R-wave morphology in V1, and the stimulus-LVAT interval (S-LVAT) was measured. QRS duration was measured from onset of first deflection, excluding the pace spike. LVAT was measured as the interval between pacing stimulus and R-peak in lead V5.

**Figure 2:**
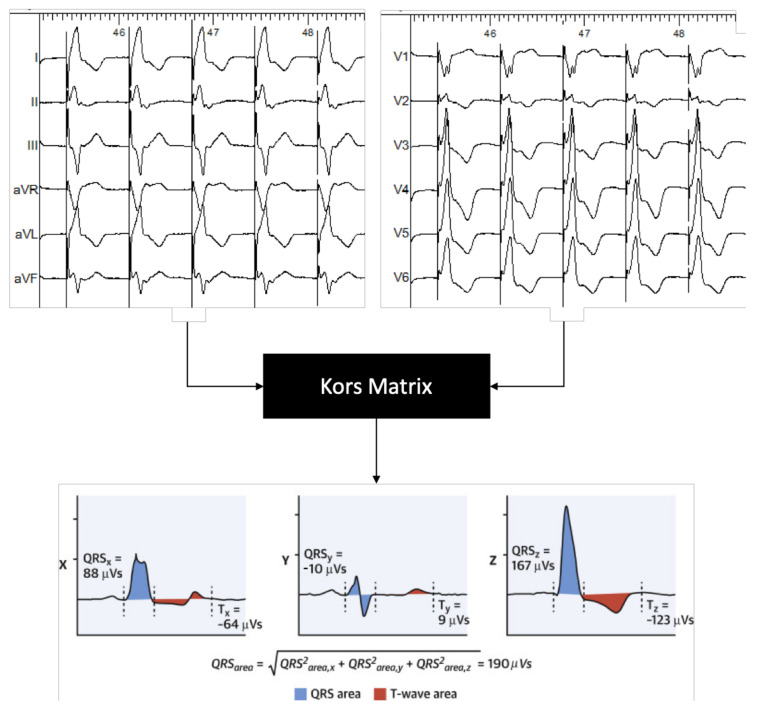
Example of a 3-dimensional vectorcardiogram (VCG) constructed from a 12-lead electrocardiogram extracted from the electrophysiology recording system.

Electrical dyssynchrony on the ventricular level was determined by converting the 2-dimensional ECG into a 3-dimensional vectorcardiogram (VCG). The VCG was synthesized as described previously [6,10]. In brief, the original digital signals were extracted from the ECG files stored in the Bard system. Subsequently, custom MATLAB software (MathWorks Inc, Natick, MA) was used to convert the 12-lead ECG into the 3 orthogonal vectorcardiography leads (X, Y, and Z) using the Kors conversion matrix, as shown in Figure 2 [11]. QRS area was calculated as the sum of the area under the QRS complex in the calculated vectorcardiographic X, Y, and Z lead (QRS area = (QRS_area,x_^2^ + QRS_area,y_^2^ + QRS_area,z_^2^)^1/2^).

### 2.5. Data Collection and Analysis

Demographic data and medical history of all patients were collected at enrollment. Procedure related characteristics, including ECG and intracardiac EGM pattern, LV peak activation time, the pacing spike-QRS interval, His-QRS interval, LBB potential-QRS interval, fluoroscopy exposure time, and doses, were recorded during implantation. Pacing electrical parameters (pacing threshold, lead impedance, and R-wave amplitude) were measured during and 1-day post-implantation. 

For post-procedural ECG and VCG analysis, 3-dimensional vectorcardiograms (VCGs) were synthesized from the recorded 12-lead ECGs using the Kors matrix [11]. To this purpose, 30 -s recordings were extracted from the electrophysiology (EP) recording system, and ectopic ventricular beats were excluded. VCG parameters, including QRS area and Azimuth, were calculated using customized software programmed in MATLAB (MathWorks, Natick, MA, USA).

### 2.6. Statistical Analysis

The number and percentage were used as descriptive statistics for categorical variables. Continuous variables were expressed as mean ± standard deviation. Differences between 2 groups were compared using the Student t-test for continuous variables. The paired t test was used to compare the differences between 2 means within the same group. Comparisons among ≥3 pacing conditions within individuals were made using repeated measures ANOVA with Bonferroni multiple comparisons procedure applied to pairwise comparisons. A 2-sided *p* value of < 0.05 was considered statistically significant. All statistical analyses were performed using SPSS Statistics version 25.0 (IBM, Chicago, IL, USA).

## 3. Results

A total of 50 patients who underwent pacemaker implantation with LBBP were prospectively included in the present study. Patient characteristics are summarized in Table 1. The patient cohort was predominantly male (61%), with a LV ejection fraction (LVEF) of 57 ± 7%. Two-thirds of patients had normal ventricular activation with an average QRS duration of 95 ± 13 ms.

### 3.1. Procedure-Related Measurements

In patients with normal ventricular activation, a LBB potential (LBB_pot_) was observed in 37/50 patients (74%) with an average LBB_pot_-QRS interval of 23 ± 8 ms. LBB capture was achieved in 31/50 patients (62%). On average, the delay between pacing stimulus and LVAT was 78 ± 11 ms.

Unipolar post-procedural (1-day follow-up) LBBP pacing threshold, pacing impedance and sensing values were 0.65 ± 0.30 V, 618 ± 225 Ω, and 13 ± 7 mV, respectively.

### 3.2. Electrophysiological Effects of LBBAP

**Figure 3:**
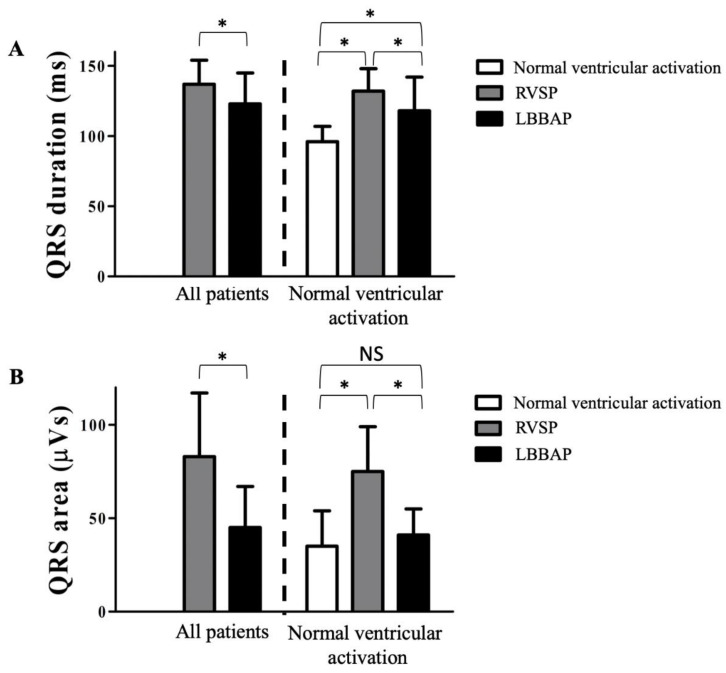
QRS duration (**A**) QRS duration in milliseconds as measured by vectorcardiogram (VCG) analysis during right ventricular septal pacing (RVSP) and Left bundle branch area pacing (LBBAP) (final lead position). In the subpopulation with normal ventricular activation, the QRS duration is also measured during intrinsic rhythm (no pacing). QRS area (**B**) QRS area in microvolt seconds as measured by VCG analysis during RVSP and LBBAP (final lead position). In the subpopulation with normal ventricular activation, the QRS duration is also measured during intrinsic rhythm (no pacing). * *p* < 0.05. NS: non-significant.

Compared to RVSP, LBBAP (all patients) significantly shortened QRS duration from 148 ± 17 ms to 123 ± 22 ms (Figure 3A). In patients with normal ventricular activation (*n* = 37), QRS duration significantly increased from 96 ± 11 ms to 142 ± 6 ms during RVSP and decreased, subsequently, to 118 ± 24 ms during LBBAP. 

For the whole group, QRS area decreased significantly from 83 ± 34 µVs during RVSP to 45 ± 22 µVs during LBBAP. In the subgroup of patients with normal ventricular activation, QRS area increased from 35 ± 19 µVs during intrinsic ventricular activation to 75 ± 24 µVs during RVSP and decreased during LBBAP to 41 ± 14 µVs (Figure 3B).

**Figure 4:**
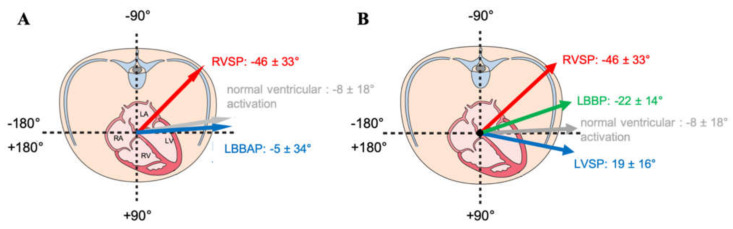
Schematic view of the transversal plane. (**A**): the angle of the QRS vector in this plane (“Azimuth”) is depicted for normal ventricular activation (in grey), RVSP (in red), and LBBAP (in blue). (**B**): Azimuth normal ventricular activation (in grey), RVSP (in red), and discriminated between LBBP (in green) and left ventricular septal pacing (LVSP) (in blue).

Beyond QRS duration and QRS area, the QRS vector in the transverse plane (Azimuth) was also analyzed. As shown in Figure 4A, LBBAP normalizes the Azimuth when compared to RVSP. Between LVSP and LBBP, there was as significant difference in Azimuth 19 ± 16° and −22 ± 14°, respectively, Figure 4B), both not significantly different from normal ventricular activation (−8 ± 18°).

### 3.3. Electrical Characteristics of LVSP and LBBP

**Figure 5:**
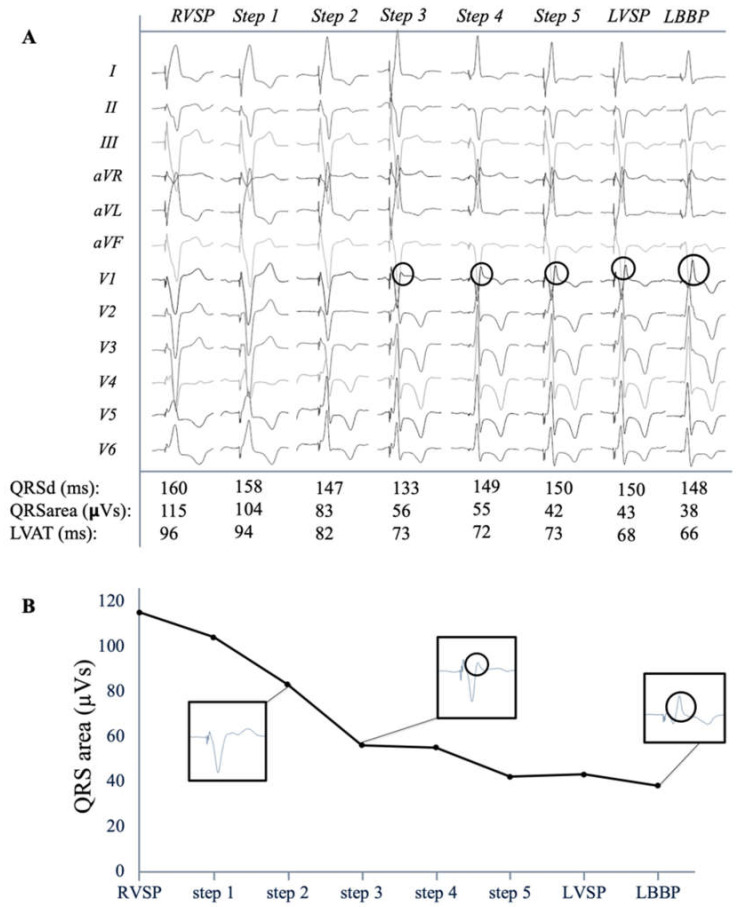
QRS morphology transition (**A**) Typical example of the 12-lead electrocardiogram of each step, from right (RVSP) to left, through the interventricular septum with selective left bundle branch pacing (LBBP) being the final step. QRS duration, area, and stimulus-left ventricular activation time (LVAT) are given. Transseptal decrease in QRS area (**B**) Decrease in QRS area for different QRS morphologies. R’/R’ is indicated with circle. QRSd: QRS duration.

The paced QRS duration, morphology and QRS area were assessed during step-by-step screwing from the right to the left side of the IVS. Figure 5A shows a typical example of the transition of the QRS complex when pacing the lead at various IVS depths. In this example QRS area gradually decreased from 115 µVs during RVSP to 38 µVs during selective LBBP. When r’ became apparent in lead V1, QRS area had largely decreased to 55 µVs. During the final few steps towards the left side of the IVS, the QRS area further decreased from 55 µVs at first visible r’ to 43 µVs during LVSP. In the final step, QRS area even further decreased to 38 µVs during LBBP. This typical example shows the additional small improvement in ventricular synchrony when LBB capture is obtained. Figure 5B illustrates that the largest reduction in QRS area is obtained when an r’ becomes present in lead V1.

**Figure 6:**
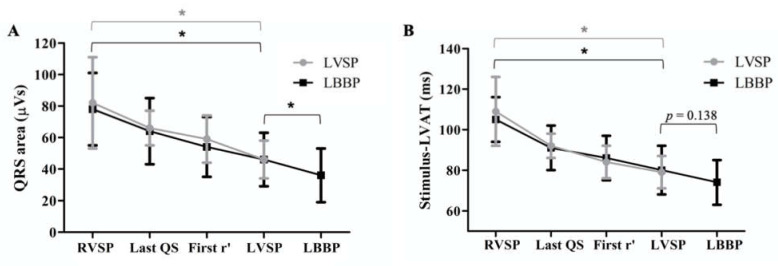
QRS area (**A**) Average absolute QRS area values for patients with (LBBP; in black) and without (LVSP; in grey) left bundle branch capture. LVAT (**B**) Average absolute stimulus-LVAT intervals for patients with (LBBP; in black) and without (LVSP; in grey) left bundle branch capture. Steps through the interventricular septum were grouped according to QRS morphology. *p < 0.05.

The change in QRS area for all patients is shown in Figure 6A, differentiated between patients where capture of the left bundle was achieved (LBBP; in black) or not (LVSP; in grey). Steps were grouped according to the following QRS morphologies: RVSP (initial pacing site), the last position with QS morphology, the first position with r’, LVSP and—in patients where LBB capture was achieved—LBBP. In patients where no LBB capture was achieved (LVSP group), QRS area significantly decreased from 82 ± 29 µVs during RVSP to 46 ± 12 µVs during LVSP. In patients where LBB capture was achieved, QRS area significantly decreased from 78 ± 23 µVs during RVSP to 46 ± 17 µVs during LVSP and further to 38 ± 15 µVs during LBBP.

In the subgroup of patients with normal ventricular activation where LBBP was achieved (*n* = 20), QRS area during LBBP was not significantly different from normal intrinsic ventricular activation (37 ± 16 vs. 35 ± 19 µVs, respectively), while QRS during LVSP was significantly larger compared to normal intrinsic ventricular activation (48 ± 17 vs. 35 ± 19 µVs, respectively).

Compared to RVSP, LBBP significantly decreased S-LVAT from 105 ± 11 ms to 74 ± 11ms. LVSP decreased S-LVAT from 109 ± 14 ms during RVSP to 81 ± 9ms. In patients where LBB capture was achieved, S-LVAT was similar between LBBP and LVSP (73 ± 15 vs. 81 ± 13 ms, *p* = 0.138), as shown in Figure 6B. Overall, there was a moderate correlation between S-LVAT and QRS area (Spearman’s *R* = 0.58, *p* < 0.05). 

## 4. Discussion

To our knowledge, this is the first study investigating the electrophysiological effects of LBBAP during the course of the lead penetrating the IVS. The primary results of this study show that, among patients with a bradycardia indication for pacing therapy, QRS area and QRS vector normalize during LBBAP. Furthermore, when the results were evaluated for capture of the left bundle, LBBP produces a significantly lower QRS area as compared to LVSP, although absolute differences are small. QRS area of the final lead position correlates moderately with LVAT and the largest decrease is achieved at first few steps penetrating the septum. Finally, the presence of r’ in lead V1 can be used to guide lead implantation achieving a significant lower QRS area.

### 4.1. QRS Duration in LBBAP

Compared to normal ventricular activation, QRS duration was significantly increased by RV pacing in our study population. This is in agreement with studies investigating RV pacing both in patients with normal cardiac function and in patients with heart failure. This emphasizes the need for replacement of the RV as standard lead implantation site, since especially pacing the RV will prolong QRS duration [12] and patients with RV paced QRS duration ≥150 milliseconds are of increased risk of developing pacing-induced cardiomyopathy [13,14]. RV pacing can eventually lead to adverse cardiac remodeling increasing the risk of atrial fibrillation (AF), heart failure, and cardiovascular death [15,16]. The present study showed that QRS duration was significantly shorter during LBBAP as compared to RV pacing, which is in line with previous studies investigating LBBAP [5,17]. To further explore the relationship between IVS pacing location and QRS duration, a subgroup of our cohort with normal ventricular activation was evaluated. In these patients, QRS duration was significantly increased during LBBAP compared to intrinsic ventricular activation, which is likely due to capture of local myocardium and delayed activation of the RV that becomes present on the ECG as a right bundle branch block pattern during LBBP. This increase in QRS duration in LBBAP was also found in other studies [4].

### 4.2. QRS Morphology in LBBAP

Beyond QRS duration, the paced morphology of the QRS complex was evaluated during lead advancement through the IVS. We found that the largest reduction in QRS area was achieved when the r’ became visible in lead V1. Further advancement of the lead, usually resulting in further increase in amplitude of r’, only resulted in a relatively small further decrease of QRS area. This might imply that QRS morphology, especially in lead V1, might be used as guidance for LBBP lead placement. The presence of an R’ in V1 during LBBAP lead implantation is illustrative for LVSP and further advancement with the goal of reaching LBB capture should be performed more carefully avoiding perforation into the LV cavity. In a small number of patients, r’ will not became visible and paced morphology will exhibit a QS pattern, potentially caused by hypertrophic or dilated LVs with impaired intraventricular conduction [18]. Determining the exact depth within the septum remains difficult, since it is usually not possible to perform ventricular pacing during screwing with conventional connector cables and the exact penetration depth is unclear from just the fluoroscopic images. However, Jastrebski et al. [19] has recently shown that the ventricular ectopy that becomes apparent as a result of screwing is present in 96% of the cases and that these so-called fixations beats are identical to the paced QRS morphology. Therefore, these fixation beats can help to identify the depth of the LBBP lead and appearance of the r’ morphology can be interpreted as a warning sign that the left side of the interventricular septum is reached. Discrimination between LBBP with and without LBB capture is now primarily bases on electrocardiographic criteria, such as QRS morphology, LVAT, and the presence LBB potential. We are in need of prospective evaluations of LBB capture.

The QRS morphology is also helpful in case septal fibrosis or scar prevents the ventricular lead to be advanced further through the septum towards the left conduction system. If r’ becomes apparent in V1, which is suggestive for deep LV septal pacing, this study shows that there is already significant improvement in electrical dyssynchrony when compared to RV septal pacing. The latter would be of particular convenience when in doubt on when to accept the LBBP lead position or when to go for a new attempt to penetrate the IVS.

Finally, using the morphology of the QRS complex to guide the LBBP lead implantation would be of particular interest for centers without an advanced electrophysiological recording system. These centers especially need the QRS morphology guidance to determine the depth of the LBBP lead and simple tools, such as the presence of R’ in V1, can be very helpful.

### 4.3. QRS Area as Measurement for Ventricular Synchrony

Our group previously proposed QRS area as measure for electrical (dys)synchrony [6], since high values of this parameter indicates dyssynchronous electrical activation, even independent of the QRS morphology [7]. In addition, QRS area has also been shown to have a strong association with clinical and echocardiographic response to CRT [8]. More recently, Ghossein et al. showed that the decrease in QRSarea due to CRT is a strong independent predictor of echocardiographic and clinical CRT response [20]. In the present study, QRS area also correlated significantly with LVAT, and Figure 5 illustrates that the transseptal behavior of QRS area and LVAT are very similar, altogether validating QRS area as measurement for ventricular synchrony.

### 4.4. QRS Area in LBBAP

The results of the present study show that in line with QRS duration, QRS area is significantly lower during LBBP as compared to RVSP. In patients with normal ventricular activation, QRS area during LBBP was even close to values of the intrinsic QRS, which indicates that LBBP maintains ventricular synchrony at a level close to normal. This is in agreement with previous studies demonstrating that LBBP maintains ventricular synchrony at levels comparable to His bundle pacing (HBP) and even to intrinsic ventricular activation [5,21,22,23]. Since pacing induced electrical dyssynchrony is minimal in LBBP, LV function is hypothesized to be preserved in patients with underlying narrow QRS complex. 

During deep septal pacing without evidence of LBB capture (defined as LVSP), QRS area was somewhat higher compared to LBBP, but the absolute difference was small. This confirms our hypothesis that the greatest reduction in dyssynchrony is already achieved by pacing subendocardially on the left side of the septum. Although the additional effort of attempting LBBP can lead to further narrowing of the QRS area, the latter might justify the choice to leave the lead when the septum is difficult to penetrate further.

### 4.5. Study Limitations

Our study should be interpreted in light of several methodologic limitations. First, this was a prospective study from two centers with a limited number of patients. Further, the exact location of pacing in the conduction system with LBBP is often hard to determine, and validated LBB capture criteria are lacking. Finally, while QRS area has been shown to have a strong association with clinical and echocardiographic response to CRT, this is not yet demonstrated for bradycardia patients.

A prospective comparison between LVSP and LBBP is needed with broad patient populations for pacing therapy, investigating electrophysiological effects—and preferably different measurements of electrical dyssynchrony, such as QRS area, LVAT, and SDAT [24], or measurements obtained with ultra-high frequency ECG (eDYS) [25], as well as mechanical effects, such as systemic or LV pressure and echocardiographic response.

## 5. Conclusions

Electrocardiographic and vectorcardiographic indices demonstrate that ventricular activation is more synchronous during both LBBP and LVSP compared to RVSP and close to values during normal intrinsic ventricular activation. Electrical synchrony is similar but slightly better during LBBP compared to LVSP.

## Figures and Tables

**Table 1:** Characteristics of patient cohort used for analysis.

Characteristics (*n* = 50)	Mean ± SD or %.
**Male sex**	61%
**Age (years)**	74 ± 10
**Medical history**	
Hypertension	61%
Atrial fibrillation	44%
Coronary artery disease	37%
Myocardial infarction	17%
**Echocardiographic parameters**	
LVEF (%)	57 ± 7
**LV end diastolic diameter (mm)**	51 ± 7
**LV end systolic diameter (mm)**	36 ± 8
IVS thickness (mm)	9 ± 1
**Electrocardiographic parameters**	
Heart rate (bpm)	66 ± 21
QRS duration (ms)	
all patients	113 ± 29
normal ventricular activation	95 ± 13
Other (escape, LBTB/RBTB)	141 ± 25
**Pacemaker indication**	
Sinus bradycardia	16%
Bradycardia-tachycardia syndrome	12%
3rd degree AV-block	35%
Ablate and pace	10%
Other	27%

## Data Availability

Data available on request due to restrictions e.g., privacy or ethical.
